# Supplementary material for: Resolving the full spectrum of human genome variation using Linked-Reads
Source: Genome Res. 2019 Apr;29(4):635–45. doi: 10.1101/gr.234443.118 (PMC6442396; doi:10.1101/gr.234443.118)
Supplement: Supplemental Material [file supp_gr.234443.118_Supplemental_longranger-2.2.zip › longranger-2.2.tar.bz2!/lr2.2-public/lib/stan/cmdstan/stan/doc/api/html/expression__grammar__def_8hpp_source.html]

Stan: src/stan/gm/grammars/expression\_grammar\_def.hpp Source File


|  |  |
| --- | --- |
|  | Stan  2.5.0  probability, sampling & optimization |


- Main Page
- Related Pages
- Namespaces
- Classes
- Files

- File List
- File Members

All Classes Namespaces Files Functions Variables Typedefs Enumerations Enumerator Friends Macros Pages

- src
- stan
- gm
- grammars

expression\_grammar\_def.hpp

Go to the documentation of this file.

1 #ifndef STAN\_\_GM\_\_PARSER\_\_EXPRESSION\_GRAMMAR\_DEF\_\_HPP

2 #define STAN\_\_GM\_\_PARSER\_\_EXPRESSION\_GRAMMAR\_DEF\_\_HPP

3

4 #include <cstddef>

5 #include <iomanip>

6 #include <iostream>

7 #include <istream>

8 #include <map>

9 #include <set>

10 #include <sstream>

11 #include <string>

12 #include <utility>

13 #include <vector>

14 #include <stdexcept>

15

16 #include <boost/spirit/include/qi.hpp>

17 // FIXME: get rid of unused include

18 #include <boost/spirit/include/phoenix\_core.hpp>

19 #include <boost/spirit/include/phoenix\_function.hpp>

20 #include <boost/spirit/include/phoenix\_fusion.hpp>

21 #include <boost/spirit/include/phoenix\_object.hpp>

22 #include <boost/spirit/include/phoenix\_operator.hpp>

23 #include <boost/spirit/include/phoenix\_stl.hpp>

24

25 #include <boost/lexical\_cast.hpp>

26 #include <boost/fusion/include/adapt\_struct.hpp>

27 #include <boost/fusion/include/std\_pair.hpp>

28 #include <boost/config/warning\_disable.hpp>

29 #include <boost/spirit/include/qi.hpp>

30 #include <boost/spirit/include/qi\_numeric.hpp>

31 #include <boost/spirit/include/classic\_position\_iterator.hpp>

32 #include <boost/spirit/include/phoenix\_core.hpp>

33 #include <boost/spirit/include/phoenix\_function.hpp>

34 #include <boost/spirit/include/phoenix\_fusion.hpp>

35 #include <boost/spirit/include/phoenix\_object.hpp>

36 #include <boost/spirit/include/phoenix\_operator.hpp>

37 #include <boost/spirit/include/phoenix\_stl.hpp>

38 #include <boost/spirit/include/support\_multi\_pass.hpp>

39 #include <boost/tuple/tuple.hpp>

40 #include <boost/variant/apply\_visitor.hpp>

41 #include <boost/variant/recursive\_variant.hpp>

42

43 #include <stan/gm/ast.hpp>

44 #include <stan/gm/grammars/whitespace\_grammar.hpp>

45 #include <stan/gm/grammars/term\_grammar.hpp>

46 #include <stan/gm/grammars/expression\_grammar.hpp>

47 #include <stan/gm/grammars/expression07\_grammar.hpp>

48

49

50 namespace stan {

51

52  namespace gm {

53

54

55  // FIXME: cut and paste from term grammar, having trouble w. includes

56  struct set\_fun\_type2 {

57  template <typename T1, typename T2>

58  struct result { typedef fun type; };

59

60  fun operator()(fun& fun,

61  std::ostream& error\_msgs) const {

62  std::vector<expr\_type> arg\_types;

63  for (size\_t i = 0; i < fun.args\_.size(); ++i)

64  arg\_types.push\_back(fun.args\_[i].expression\_type());

65  fun.type\_ = function\_signatures::instance().get\_result\_type(fun.name\_,

66  arg\_types,

67  error\_msgs);

68  return fun;

69  }

70  };

71  boost::phoenix::function<set\_fun\_type2> set\_fun\_type2\_f;

72

73  struct binary\_op\_expr {

74  template <typename T1, typename T2, typename T3, typename T4, typename T5>

75  struct result { typedef expression type; };

76

77  expression operator()(expression& expr1,

78  const expression& expr2,

79  const std::string& op,

80  const std::string& fun\_name,

81  std::ostream& error\_msgs) const {

82  if (!expr1.expression\_type().is\_primitive()

83  || !expr2.expression\_type().is\_primitive()) {

84  error\_msgs << "binary infix operator "

85  << op

86  << " with functional interpretation "

87  << fun\_name

88  << " requires arguments or primitive type (int or real)"

89  << ", found left type=" << expr1.expression\_type()

90  << ", right arg type=" << expr2.expression\_type()

91  << "; ";

92  }

93  std::vector<expression> args;

94  args.push\_back(expr1);

95  args.push\_back(expr2);

96  set\_fun\_type2 sft;

97  fun f(fun\_name,args);

98  sft(f,error\_msgs);

99  return expression(f);

100  }

101  };

102  boost::phoenix::function<binary\_op\_expr> binary\_op\_f;

103

104

105  template <typename Iterator>

106  expression\_grammar<Iterator>::expression\_grammar(variable\_map& var\_map,

107  std::stringstream& error\_msgs)

108  : expression\_grammar::base\_type(expression\_r),

109  var\_map\_(var\_map),

110  error\_msgs\_(error\_msgs),

111  expression07\_g(var\_map,error\_msgs,\*this)

112  {

113  using boost::spirit::qi::\_1;

114  using boost::spirit::qi::char\_;

115  using boost::spirit::qi::double\_;

116  using boost::spirit::qi::eps;

117  using boost::spirit::qi::int\_;

118  using boost::spirit::qi::lexeme;

119  using boost::spirit::qi::lit;

120  using boost::spirit::qi::\_pass;

121  using boost::spirit::qi::\_val;

122  using boost::spirit::qi::labels::\_r1;

123

124  // \_r1 : var\_origin

125

126  expression\_r.name("expression (top level, precedence 15, binary ||");

127  expression\_r

128  = expression14\_r(\_r1) [\_val = \_1]

129  > \*( lit("||")

130  > expression14\_r(\_r1) [\_val = binary\_op\_f(\_val,\_1,"||","logical\_or",

131  boost::phoenix::ref(error\_msgs))]

132  );

133

134  expression14\_r.name("expression, precedence 14, binary &&");

135  expression14\_r

136  = expression10\_r(\_r1) [\_val = \_1]

137  > \*( lit("&&")

138  > expression10\_r(\_r1) [\_val = binary\_op\_f(\_val,\_1,"&&","logical\_and",

139  boost::phoenix::ref(error\_msgs))]

140  );

141

142  expression10\_r.name("expression, precedence 10, binary ==, !=");

143  expression10\_r

144  = expression09\_r(\_r1) [\_val = \_1]

145  > \*( ( lit("==")

146  > expression09\_r(\_r1) [\_val = binary\_op\_f(\_val,\_1,"==","logical\_eq",

147  boost::phoenix::ref(error\_msgs))] )

148  |

149  ( lit("!=")

150  > expression09\_r(\_r1) [\_val = binary\_op\_f(\_val,\_1,"!=","logical\_neq",

151  boost::phoenix::ref(error\_msgs))] )

152  );

153

154  expression09\_r.name("expression, precedence 9, binary <, <=, >, >=");

155  expression09\_r

156  = expression07\_g(\_r1) [\_val = \_1]

157  > \*( ( lit("<=")

158  > expression07\_g(\_r1) [\_val = binary\_op\_f(\_val,\_1,"<","logical\_lte",

159  boost::phoenix::ref(error\_msgs))] )

160  |

161  ( lit("<")

162  > expression07\_g(\_r1) [\_val = binary\_op\_f(\_val,\_1,"<=","logical\_lt",

163  boost::phoenix::ref(error\_msgs))] )

164  |

165  ( lit(">=")

166  > expression07\_g(\_r1) [\_val = binary\_op\_f(\_val,\_1,">","logical\_gte",

167  boost::phoenix::ref(error\_msgs))] )

168  |

169  ( lit(">")

170  > expression07\_g(\_r1) [\_val = binary\_op\_f(\_val,\_1,">=","logical\_gt",

171  boost::phoenix::ref(error\_msgs))] )

172  );

173

174  }

175  }

176 }

177

178 #endif

stan::gm::binary\_op\_expr::result::type

expression type

**Definition:** expression\_grammar\_def.hpp:75

stan::gm::set\_fun\_type2\_f

boost::phoenix::function< set\_fun\_type2 > set\_fun\_type2\_f

**Definition:** expression\_grammar\_def.hpp:71

stan::gm::fun::name\_

std::string name\_

**Definition:** ast.hpp:351

stan::gm::fun::type\_

expr\_type type\_

**Definition:** ast.hpp:353

expression07\_grammar.hpp

stan::gm::function\_signatures::instance

static function\_signatures & instance()

**Definition:** ast\_def.cpp:143

stan::gm::binary\_op\_f

boost::phoenix::function< binary\_op\_expr > binary\_op\_f

**Definition:** expression\_grammar\_def.hpp:102

stan::gm::set\_fun\_type2::result::type

fun type

**Definition:** expression\_grammar\_def.hpp:58

expression\_grammar.hpp

stan::gm::expression\_grammar

**Definition:** expression07\_grammar.hpp:23

stan::gm::expression\_grammar::expression09\_r

boost::spirit::qi::rule< Iterator, expression(var\_origin), whitespace\_grammar< Iterator > > expression09\_r

**Definition:** expression\_grammar.hpp:52

stan::gm::fun::args\_

std::vector< expression > args\_

**Definition:** ast.hpp:352

stan::gm::expression\_grammar::expression07\_g

expression07\_grammar< Iterator > expression07\_g

**Definition:** expression\_grammar.hpp:41

stan::gm::set\_fun\_type2::result

**Definition:** expression\_grammar\_def.hpp:58

stan::gm::expression\_grammar::expression14\_r

boost::spirit::qi::rule< Iterator, expression(var\_origin), whitespace\_grammar< Iterator > > expression14\_r

**Definition:** expression\_grammar.hpp:62

stan::gm::variable\_map

**Definition:** ast.hpp:439

stan::gm::binary\_op\_expr

**Definition:** expression\_grammar\_def.hpp:73

stan::gm::expression

**Definition:** ast.hpp:216

term\_grammar.hpp

stan::gm::binary\_op\_expr::result

**Definition:** expression\_grammar\_def.hpp:75

ast.hpp

stan::gm::set\_fun\_type2

**Definition:** expression\_grammar\_def.hpp:56

stan::gm::fun

**Definition:** ast.hpp:350

stan::gm::set\_fun\_type2::operator()

fun operator()(fun &fun, std::ostream &error\_msgs) const

**Definition:** expression\_grammar\_def.hpp:60

whitespace\_grammar.hpp

stan::gm::expression\_grammar::expression\_r

boost::spirit::qi::rule< Iterator, expression(var\_origin), whitespace\_grammar< Iterator > > expression\_r

**Definition:** expression\_grammar.hpp:47

stan::gm::expression\_grammar::expression10\_r

boost::spirit::qi::rule< Iterator, expression(var\_origin), whitespace\_grammar< Iterator > > expression10\_r

**Definition:** expression\_grammar.hpp:57

stan::gm::binary\_op\_expr::operator()

expression operator()(expression &expr1, const expression &expr2, const std::string &op, const std::string &fun\_name, std::ostream &error\_msgs) const

**Definition:** expression\_grammar\_def.hpp:77

stan::gm::expression\_grammar::expression\_grammar

expression\_grammar(variable\_map &var\_map, std::stringstream &error\_msgs)

**Definition:** expression\_grammar\_def.hpp:106

stan::gm::function\_signatures::get\_result\_type

expr\_type get\_result\_type(const std::string &name, const std::vector< expr\_type > &args, std::ostream &error\_msgs)

**Definition:** ast\_def.cpp:329

stan::gm::expression::expression\_type

expr\_type expression\_type() const

**Definition:** ast\_def.cpp:570

stan::gm::expr\_type::is\_primitive

bool is\_primitive() const

**Definition:** ast\_def.cpp:93


---

[ Stan Home Page ]

*© 2011–2014,
Stan Development Team.*
